# Analysis of the outcomes of postdiverticulitis investigations: a multicentre cohort study including 1,120 patients

**DOI:** 10.1308/rcsann.2024.0077

**Published:** 2024-10-09

**Authors:** A Abdelrahim, O Ali, D Kamali, A Reddy, S Harrison, M Boshnaq, H Abudeeb, F Ashoush, M Qulaghassi, S Eldesouky, M Mansour, SF Rahman-Casans, K Osman

**Affiliations:** ^1^ Health Education England Northeast, UK; ^2^ East Lancashire Hospitals NHS Foundation Trust, UK; ^3^ County Durham and Darlington NHS Foundation Trust, UK; ^4^ South Tees Hospitals NHS Trust, UK; ^5^ East Kent Hospitals NHS Foundation Trust, UK; ^6^ Ain Shams University, Egypt; ^7^ Gateshead Healthcare NHS Foundation Trust, UK

**Keywords:** Diverticulitis, Colonic, Hinchey

## Abstract

**Introduction:**

The aim of this study was to assess the yield of the endoscopic investigations performed following the resolution of acute diverticulitis.

**Methods:**

A retrospective multicentre study included patients with multislice computed tomography (MSCT)-proven diverticulitis, in four NHS hospitals, between January 2016 and April 2023. The primary outcome was the rate of colonic cancer in the diseased segment. Secondary outcomes included the rate of malignancy in the nondiseased colonic segments, the benign colonic polyp detection rate, the rate of malignancy in the resected surgical specimens in patients who underwent an emergency surgery on the index admission and the rate of complications in the investigated group.

**Results:**

A total of 1,120 patients were included in the study, out of which 604 were females, with a median age of 61 years; 731 patients (65%) had uncomplicated diverticulitis (Hinchey 1A) while 389 (35%) had complicated diverticulitis (Hinchey 1B-4). Following the acute episode, 757 (74%) patients had subsequent endoscopic evaluation. The incidence of colorectal cancer (CRC) or advanced adenomas (AA) in patients with uncomplicated diverticulitis was 0.14%. In the complicated diverticulitis group, the incidence of CRC/AA in patients with Hinchey 1b and Hinchey 2 was 1.4% and 5.4%, respectively. Out of the 107 patients who underwent emergency colonic resection for suspected perforated diverticulitis, 18 (16.8%) had histological evidence of colonic malignancy.

**Conclusions:**

Endoscopic investigations following uncomplicated diverticulitis have a low yield for sinister colonic pathology. Colonoscopy should be planned following complicated diverticulitis and in patients with uncomplicated diverticulitis with suspicious radiological finding on index imaging or in patients with ongoing clinical manifestations. In patients who undergo emergency surgery, oncological principles should be applied whenever possible.

## Introduction

Colonic diverticulosis is a common disease in the UK and Western countries. The incidence of diverticulosis increases with age, with 85% of patients diagnosed with diverticulosis aged 50 years or more.^1^ It is estimated that 10% of the population aged 45 years and 80% of those aged 80 years have evidence of diverticulosis. Patients with diverticulosis have a 10–25% risk developing diverticulitis during their lifetime.^2^ Diverticular disease distribution is more common in the sigmoid and left colon where stools become harder and more formed; however, pancolonic diverticular disease may also develop. Right-sided colonic diverticulosis is encountered primarily in elderly patients with pandiverticulosis; racial variation has been reported, with right colonic diverticulosis more prevalent in Asian patients.^3^ Approximately 80–88% of patients with acute diverticulitis are treated nonoperatively.^4^

Previous national and international guidelines suggested routine colonic evaluation by endoscopy or computed tomography (CT) colonography following the resolution of acute diverticulitis to rule out underlying malignancy or inflammatory bowel disease.^5–7^ However, these recommendations were weak and based on expert opinions lacking high-quality data. More recent guidelines advise that endoscopic evaluation following acute diverticulitis is indicated only in patients with high-risk features.^8–10^

Our study aims to determine the yield of endoscopic investigations following the management of CT-proven acute diverticulitis. The primary outcome of the study was the rate of malignancy detected by subsequent investigations in the diseased colonic segment. The secondary outcomes were the rate of malignancy in the nondiseased colon, the benign colonic polyp detection rate, the rate of malignancy in the resected surgical specimens of the patients who underwent an emergency Hartmann's procedure on the index admission and the incidence rate of complications in the investigated group of patients.

### Study design and setting

This is a retrospective multicentre cohort study that took place across four hospitals (three busy district general hospitals and one tertiary centre) in England. The inclusion criteria were as follows: all patients who presented with CT-proven acute diverticulitis to Queen Elizabeth the Queen Mother Hospital, Margate between January 2016 and December 2018, Royal Blackburn Hospital between January 2019 and December 2020, James Cook University Hospital between January 2021 and December 2022 and Darlington Memorial Hospital between January and April 2023. The exclusion criteria consisted of patients who had small bowel or appendiceal diverticulitis and those with a clinical diagnosis of diverticulitis that was not proven or investigated by CT imaging (Hinchey 0).

### Participants and procedure

The hospitals' coding systems including PACS (Picture Archiving and Communication Systems), endoscopy database and daily General Surgery handover sheets were used to identify the patients who were reviewed or admitted by the surgical team with a diagnosis of diverticulitis. Only the patients who had a CT-proven acute colonic diverticulitis were included in our study. The severity of inflammation was classified using the modified Hinchey's classification (Table 1).

**Table 1:** Modified Hinchey classification

Modified Hinchey classification	CT findings
Hinchey 0	Mild clinically diagnosed diverticulitis (no CT scan)
Hinchey 1a	Pericolic fat stranding/phlegmon
Hinchey 1b	Pericolic abscess formation
Hinchey 2	Pelvic intra-abdominal/retroperitoneal abscess
Hinchey 3	Generalized purulent peritonitis
Hinchey 4	Generalized faecal peritonitis

CT = computed tomography

### Data collection and analysis

Data collection began at least three months after the end of the specified period for each hospital. This was to allow enough time to investigate all the subsequent investigations and findings for all the patients involved in the study. Data were collected in a coded Excel spreadsheet. Data included socio-demographic (age, gender), previous episodes of diverticulitis, modality of treatment (including antibiotics, radiologically guided drainage, surgery or an end-of-life pathway), length of hospital stay, history and findings of previous endoscopy, subsequent investigations following discharge and their findings.

The SPSS 27 statistics software (IBM Corp., Chicago, IL, USA) was used to conduct the statistical analysis. Categorical and continuous variables were described in the text in the forms of means and ranges as well as depicted in figures and crosstabs. Correlations were made between the independent variables and findings. Furthermore, categorical variables were further examined using chi-squares. A *p* value of <0.05 demonstrated statistical significance.

## Results

**Figure 1:**
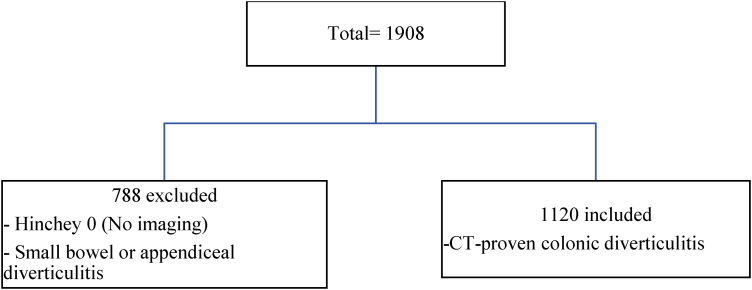
Total number of included and excluded patients.CT = computed tomography

A total of 1,908 patients were identified to have been admitted with diverticulitis during the specified periods (Figure 1). In all, 788 patients were excluded from the study, leaving 1,120 patients included in the study, out of which 604 (54%) were female. The median age of patients was 61 years, ranging between 21 and 95 years. A total of 418 patients (37.3%) had a previous episode(s) of diverticulitis before the index admission; 731 patients (65.2%) had uncomplicated (Hinchey 1a) diverticulitis, 145 patients (13%) had a pericolic abscess (Hinchey grade 1b), whereas 112 patients (10%) presented with a pelvic abscess (Hinchey grade 2) and 132 patients (11.8%) presented with generalized peritonitis (Hinchey grade 3 or 4) (Table 2).

**Table 2:** Patient demographics and details of index admissions

	*n* (%)
Age	
50 years and above	851 (75.9%)
Below 50 years	269 (24%)
Sex	
Female	604 (54%)
Male	516 (46%)
Previous episodes of diverticulitis	
No/unknown	702 (63%)
Yes	418 (37%)
Hinchey stage	
1a	731 (65.2%)
1b	145 (13%)
2	112 (10%)
3/4	132 (11.8%)
Management of index admission	
Antibiotics only	890 (79.5%)
Emergency Hartmann's procedure	107 (9.5%)
Image-guided drainage	61 (5.5%)
Washout with or without stoma	30 (2.7%)
EOLCP	32 (2.8%)

EOLCP = end-of-life care pathway

Most cases (896 patients, 80%) were managed conservatively with oral or intravenous antibiotics, out of which 113 patients did not require admission to hospital and were managed through the Same Day Emergency Care Unit (SDEC) pathway. A total of 61 patients (5.4%) required a radiology-guided drainage, 30 patients (2.6%) had a laparoscopic or open washout with or without a colostomy and 107 patients (9.5%) underwent an emergency resection (Hartmann's procedure). None of the surgical management group had a primary anastomosis during the index surgery. A total of 32 patients (2.8%) were put on the End-of-Life care pathway (EOLCP) and died during the index admission (Table 2). Our data are comparable with the findings of the DAMASCUS study (Diverticulitis Management, a Snapshot Collaborative Audit Study); however, none of the four investigated sites were involved in this study during the window of our data collection.^11^ The details of outcomes of patients' treatment and progress during the index admissions or further admissions were not discussed in detail to avoid complicating the study results as this was not the main aim of the study.

Following the resolution of the acute episode, 757 patients were sent for colonic visualisation, out of which 542 (71.5%) were from the Hinchey 1a group, 123 (16.2%) from the Hinchey 1b group, 87 (11.4%) from the Hinchey 2 group and 5 (0.6%) from the Hinchey 3/4 group who underwent washout only. A total of 426 patients (56.3%) had a colonoscopy, 282 (37.3%) had a flexible sigmoidoscopy and 49 (6.4%) had a CT colonography; 126 patients had a lower gastrointestinal endoscopy in the three years prior to their index admission. Of these 126 patients, 44 (35%) had subsequent investigations following the acute attack, out of which 26/44 had a flexible sigmoidoscopy, 15/44 had a colonoscopy and 3/44 had a CT colonography. The average time to investigation following acute diverticulitis was 41 days (∼6 weeks), ranging from 10 days to 74 days (∼11 weeks). Patients who underwent emergency Hartmann's procedures were followed up in the outpatient setting. The details of further investigations for those patients are not discussed in this manuscript.

**Figure 2:**
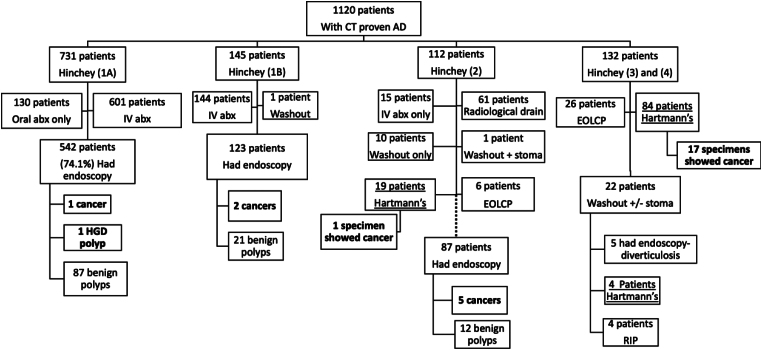
Flowchart of the overall management of all patients.abx = antibiotics; AD = acute diverticulitis; CT = computed tomography; EOLCP = end-of-life care pathway; HGD = high-grade dysplasia; IV = intravenous; RIP = died

The results revealed that four patients of the investigated cohort (total number 757) were found to have a malignant lesion in the diseased colon segment, one of which had Hinchey stage 1a, one had Hinchey stage 1b and two had Hinchey stage 2 diverticulitis on the index admission (Figure 2). Of the five patients who had colonic malignancy in the nondiseased colon, one had uncomplicated diverticulitis (Hinchey 1a) on the index admission, one had Hinchey 1b diverticulitis and the other three cancers were found in patients who had Hinchey 2 diverticulitis. The general practice records of those patients who had colonic malignancy on further investigations showed that two patients had ongoing symptoms prior to the index presentation that would have warranted a colonoscopy. A total of 121 patients had benign-looking colonic polyps, ranging between 13.8% and 20% in the four groups, which is comparable with the adenoma detection rate in our asymptomatic screening population. It is worth mentioning that 114 (94%) of these polyps were found in the nondiseased colon or rectum. Out of the 107 patients who had surgical resection (Hartmann's procedure), the histological examination of the resected segment confirmed the presence of malignancy in 18 patients (16.8%). Primary sigmoid adenocarcinoma was found in 15 cases, while one specimen showed appendiceal mucinous adenocarcinoma associated with sigmoid diverticulitis and two patients had metastatic lesions, one from a lung neuroendocrine tumour and the other had a metastatic ovarian cancer. Despite having a radiological diagnosis of diverticulitis in all those cases, coexisting diverticular disease was reported in the resected specimen of 11/18 cases. One patient had a histological diagnosis of severe ulcerative colitis along with diverticulosis. Subgroup analysis revealed no statistically significant difference between the four hospitals' cohorts of patients in relation to patients' demographics, the rate of endoscopic investigations of different groups or endoscopic findings.

Eleven patients were re-admitted following endoscopic investigations. Two patients were admitted with nonspecific abdominal pain, eight patients had postcolonoscopy bleeding and one patient had a splenic hematoma. One patient needed endoscopic clipping of a bleeding polypectomy site. All others were managed conservatively.

## Discussion

Previous national and international guidelines recommended routine luminal colonic screening by either endoscopy, barium enema or CT colonography following the resolution of acute diverticulitis.^5–7^ However, these recommendations were based on low-quality evidence. There has been no evidence of a causal link between colonic diverticular disease and malignancy. A plausible reason for this mis-association could be that the diagnosis of diverticulitis in earlier studies was based on clinical examination, imaging modalities including ultrasonography, barium enemas and lower-quality CT scans. The recent advances in the quality of CT scanners and reporting enhanced the accuracy of the diagnosis of diverticular disease with a high sensitivity and specificity of 99% and 95%, respectively.^12^

More recent national and international guidelines recommend that routine colonoscopy is no longer indicated routinely following uncomplicated diverticulitis.^8–10^ An international Delphi study in 2015 including a group of expert colorectal surgeons advised against the practice of routine colonoscopy in all patients with uncomplicated diverticulitis. The consensus opinion was that endoscopy is advised only in selected patients with high-risk features.^13^ This practice is supported by the latest guidelines produced by ACPGBI, European Society of Coloproctology (ESCP) and the World Society of Emergency Surgery (WSES), which recommend that routine interval colonoscopy should be undertaken only in patients with either complicated diverticular disease, persisting symptoms or in patients who have suspicious findings on CT scan.^8–10^

Several studies have analyzed the colon cancer detection rate in patients who underwent endoscopic investigations following acute diverticulitis. The reported incidence of colonic neoplasia varied significantly between different studies. Multiple recent meta-analyses and systematic reviews reported the rate of associated colonic malignancy between 0.25% and 0.78% in uncomplicated diverticulitis and 7.8% to 10.9% in complicated diverticulitis.^14–26^ Our research demonstrates that the incidence of colorectal cancer (CRC) or advanced adenomas (AA) in patients with uncomplicated (Hinchey 1a) diverticulitis is 0.14%. The incidence of CRC/AA in patients with Hinchey 1b and Hinchey 2 is 1.4% and 5.4%, respectively. Interestingly, 50% of diagnosed malignant lesions were detected in the nondiseased colon. CRC was detected in 16.8% of the surgical specimens of patients who underwent emergency resection.

Among all cases that had an endoscopic procedure post diverticulitis, 11 patients (1.5%) were readmitted with complications secondary to colonoscopy. This puts into question the need for an endoscopy procedure postdiverticulitis, with its attendant risks and additional costs related to procedures that may not add to optimal patient care.

In conclusion, there is a lack of robust evidence regarding the causal correlation between diverticulitis and CRC. Multiple recent studies including our study suggest that the cohort of patients who have a CT-proven uncomplicated diverticulitis may have a risk of underlying or associated CRC or AA that is equivalent to or less than the normal population risk. Therefore, those patients may follow the national bowel cancer screening pathway with no need for additional endoscopic investigations. Colonoscopy should be planned for patients who had complicated diverticulitis, Hinchey stage 1b or beyond and patients with uncomplicated diverticulitis who have high-risk features and patients whose CT scan showed a suspicion of underlying mass lesion. We suggest that patients who are referred for endoscopic investigations should have a full colonoscopy, if technically feasible, to rule out malignant lesions in the nondiseased colon. Surgeons should aim to perform an oncological colonic resection for perforated diverticulitis, as the risk of concomitant malignancy is relatively high.

## Limitations

We acknowledge some limitations to our study that might affect the generalizability of our findings. The periods investigated in the four centres were sequential rather than simultaneous based on the rotations of the main investigator of the study. The study period encompasses the COVID pandemic, which certainly had a significant impact on the management of surgical emergencies including acute diverticulitis and delay to the access for further endoscopic investigations. This might be a source of information bias in our data.

## Data Availability

The datasets generated during and/or analysed during the current study are available from the corresponding author on reasonable request.

## References

[1] Weizman AV, Nguyen GC. Diverticular disease: epidemiology and management. *Can J Gastroenterol* 2011; **25**: 385–389. 21876861 10.1155/2011/795241 PMC3174080

[2] Stollman N, Raskin JB. Diverticular disease of the colon. *Lancet* 2004; **363**: 631–639. 14987890 10.1016/S0140-6736(04)15597-9

[3] Kim JH, Cheon JH, Park S *et al.* Relationship between disease location and age, obesity, and complications in Korean patients with acute diverticulitis: a comparison of clinical patterns with those of Western populations. *Hepatogastroenterology* 2008; **55**: 983–986. 18705312

[4] Strate LL, Morris AM. Epidemiology, pathophysiology, and treatment of diverticulitis. *Gastroenterology* 2019; **156**: 1282–1298.e1. 30660732 10.1053/j.gastro.2018.12.033 PMC6716971

[5] Royal College of Surgeons (RCS) Commissioning guide: Colonic diverticular disease. https://www.rcseng.ac.uk/-/media/files/rcs/library-and-publications/non-journal-publications/colinic-diverticular-disease-commissioning-guide.pdf

[6] Stollman N, Smalley W, Hirano I *et al.* American Gastroenterological Association Institute guideline on the management of acute diverticulitis. *Gastroenterology* 2015; **149**: 1944–1949. 26453777 10.1053/j.gastro.2015.10.003

[7] Feingold D, Steele SR, Lee S *et al.* Practice parameters for the treatment of sigmoid diverticulitis. *Dis Colon Rectum* 2014; **57**: 284–294. 24509449 10.1097/DCR.0000000000000075

[8] Miller AS, Boyce K, Box B *et al.* The association of coloproctology of Great Britain and Ireland consensus guidelines in emergency colorectal surgery. *Colorectal Dis* 2021; **23**: 476–547. 33470518 10.1111/codi.15503 PMC9291558

[9] Sartelli M, Weber DG, Kluger Y *et al.* Update of the WSES guidelines for the management of acute colonic diverticulitis in the emergency setting. *World J Emerg Surg* 2020; **15**: 32. 32381121 10.1186/s13017-020-00313-4 PMC7206757

[10] Schultz JK, Azhar N, Binda GA *et al.* European society of coloproctology: guidelines for the management of diverticular disease of the colon. *Colorectal Dis* 2020; **22**: 5–28. 10.1111/codi.15140 32638537

[11] Rabie M, Fowler H, Dudi-Venkata NN *et al.* Diverticulitis management, a snapshot collaborative audit study (DAMASCUS): protocol for an international, multicentre, prospective observational study. *Colorectal Dis* 2021; **23**: 2182–2188. 33915018 10.1111/codi.15699

[12] Kircher MF, Rhea JT, Kihiczak D *et al.* Frequency, sensitivity, and specificity of individual signs of diverticulitis on thin-section helical CT with colonic contrast material: experience with 312 cases. *AJR Am J Roentgenol* 2002; **178**: 1313–1318. 12034590 10.2214/ajr.178.6.1781313

[13] O’Leary DP, Lynch N, Clancy C *et al.* International, expert-based, consensus statement regarding the management of acute diverticulitis. *JAMA Surg* 2015; **150**: 899–904. 26176318 10.1001/jamasurg.2015.1675

[14] Tehranian S, Klinge M, Saul M *et al.* Prevalence of colorectal cancer and advanced adenoma in patients with acute diverticulitis: implications for follow-up colonoscopy. *Gastrointest Endosc* 2019; **91**: 634–640. 31521778 10.1016/j.gie.2019.08.044 PMC7039754

[15] Lau KC, Spilsbury K, Farooque Y *et al.* Is colonoscopy still mandatory after a CT diagnosis of left-sided diverticulitis: Can colorectal cancer be confidently excluded? *Dis Colon Rectum* 2011; **54**: 1265–1270. 21904141 10.1097/DCR.0b013e31822899a2

[16] Soh NYT, Chia DKA, Teo NZ *et al.* Prevalence of colorectal cancer in acute uncomplicated diverticulitis and the role of the interval colonoscopy. *Int J Colorectal Dis* 2018; **33**: 991–994. 29663068 10.1007/s00384-018-3039-1

[17] Sallinen V, Mentula P, Leppäniemi A. Risk of colon cancer after computed tomography-diagnosed acute diverticulitis: is routine colonoscopy necessary? *Surg Endosc* 2014; **28**: 961–966. 24178863 10.1007/s00464-013-3257-0

[18] Sharma PV, Eglinton T, Hider P *et al.* Systematic review and meta-analysis of the role of routine colonic evaluation after radiologically confirmed acute diverticulitis. *Ann Surg* 2014; **259**: 263–272. 24169174 10.1097/SLA.0000000000000294

[19] de Vries HS, Boerma D, Timmer R *et al.* Routine colonoscopy is not required in uncomplicated diverticulitis: a systematic review. *Surg Endosc* 2014; **28**: 2039–2047. 24488358 10.1007/s00464-014-3447-4

[20] Brar MS, Roxin G, Yaffe PB *et al.* Colonoscopy following nonoperative management of uncomplicated diverticulitis may not be warranted. *Dis Colon Rectum* 2013; **56**: 1259–1264. 24105001 10.1097/DCR.0b013e3182a26bfd

[21] Suhardja TS, Norhadi S, Zeah EZ *et al.* Is early colonoscopy after CT-diagnosed diverticulitis still necessary? *Int J Colorectal Dis* 2017; **32**: 485–489. 28035461 10.1007/s00384-016-2749-5

[22] Daniels L, Ünlü Ç, de Wijkerslooth TR *et al.* Yield of colonoscopy after recent CT-proven uncomplicated acute diverticulitis: a comparative cohort study. *Surg Endosc* 2014; **29**: 2605–2613. 25472747 10.1007/s00464-014-3977-9

[23] Rottier SJ, Van Dijk ST, van Geloven AAW *et al.* Meta-analysis of the role of colonoscopy after an episode of left-sided acute diverticulitis. *Br J Surg* 2019; **106**: 988–997. 31260589 10.1002/bjs.11191 PMC6618242

[24] Westwood DA, Eglinton TW, Frizelle FA*.* Routine colonoscopy following acute uncomplicated diverticulitis. *Br J Surg* 2011; **98**: 1630–1634. 21713756 10.1002/bjs.7602

[25] Niv Y, Hazazi R, Levi Z *et al.* Screening colonoscopy for colorectal cancer in asymptomatic people: a meta-analysis. *Dig Dis Sci* 2008; **53**: 3049–3054. 18463980 10.1007/s10620-008-0286-y

[26] Azizian JM, Trieu H, Kovacs TO *et al.* Yield of post-acute diverticulitis colonoscopy for ruling out colorectal cancer. *Tech Innov Gastrointest Endosc* 2022; **24**: 254–261. 36540108 10.1016/j.tige.2022.04.001 PMC9762736

